# A Small Target Localization Method Based on the Magnetic Gradient Tensor

**DOI:** 10.3390/mi13101639

**Published:** 2022-09-29

**Authors:** Bo Wang, Guoquan Ren, Zhining Li, Qingzhu Li, Ziming Cai

**Affiliations:** Department of Vehicle and Electrical Engineering, Shijiazhuang Branch, Army Engineering University of PLA, Shijiazhuang 050003, China

**Keywords:** magnetic gradient tensor, noise, absolute error, accuracy

## Abstract

Currently, many small target localization methods based on a magnetic gradient tensor have problems, such as complex solution processes, poor stability, and multiple solutions. This paper proposes an optimization method based on the Euler deconvolution localization method to solve these problems. In a simulation, the Euler deconvolution method, an improved method of the Euler deconvolution method and our proposed method are analyzed under noise conditions. These three methods are evaluated in the field with complex magnetic interference in an experiment. The simulations show that the accuracy of the proposed method is higher than that of the improved Euler deconvolution method and is slightly lower for noisy conditions. The experimental results show that the proposed method is more precise and accurate than the Euler deconvolution and enhanced methods.

## 1. Introduction

The term “tensor” was first proposed in 1846 and widely accepted by 1915. With the introduction of tensors in the magnetic detection field, magnetic gradient tensors have become the primary detection data used in the field of modern magnetic detection. Detection accuracy has significantly improved because a magnetic gradient tensor is not affected by the geomagnetic field [1]. As technology has developed, magnetic detection technology has been widely used in the military and the medical fields, as well as terrain exploration and other fields [2,3,4,5,6,7,8,9].

The purpose of magnetic field detection is to locate a target more accurately. Many experts and scholars have carried out research in this area. Hu et al. used a magnetic field vector and magnetic gradient tensor to obtain the underwater multi-target positioning method [10]. Based on a magnetic dipole model, Zhi et al. derived a new localization method, using the geometric invariants of tensors [11] that had good stability. Yin et al. proposed a ferromagnetic target localization and a recognition method based on the rotation invariants [12]. Nara et al. proposed a new localization formula using the Euler deconvolution [13]. This method could obtain an accurate localization result in real-time. However, the method was greatly affected by the background field. Currently, the localization method proposed by Nara et al. is the simplest to solve the process. Many experts and scholars have improved upon this formula.

Based on the localization method proposed by Nara, Yin et al. took the derivative of both sides of the formula so that measuring the magnetic field vector was unnecessary [14]. However, the high-order magnetic gradient tensor used is easily affected by noise. Wang et al. proposed a method for high-order magnetic gradient tensors with higher stability, given the poor stability of high-order magnetic gradient tensors [15]. Their results showed that this method could effectively improve the stability of high-order tensors. Wang et al. enhanced the stability of magnetic gradient tensor data based on Nara’s method [16]. The localization error of this method was smaller under complex magnetic interference conditions. Xu et al. used the difference between two points to approximate the magnetic gradient tensor [17]. This method avoided measuring three components of the magnetic field. However, the forward difference method was used to approximate the tensors, which increased the error of the localization method.

Based on the method proposed by Xu et al., this paper presents a way to locate a small target using a three-point magnetic gradient tensor. The proposed method avoids measuring the three components of a magnetic field and reduces the error caused by the forward difference method.

The structure of this paper is as follows: Section 2 introduces the definition of a magnetic gradient tensor. Section 3 introduces the localization method’s specific formula and two other localization methods. Section 4 describes how the magnetic dipole is used as the model, and the simulation is carried out under noisy conditions. The absolute error and the RMSE (the root-mean-square-error) are used to evaluate the localization results. The results show that the error of this proposed method is low. Section 5 describes how experiments are conducted to verify the accuracy by comparing the localization results, the absolute error, and the RMSE (the root-mean-square-error). Section 6 summarizes the paper.

## 2. The Basic Theory

When the detection distance is much larger than the magnetic anomaly source, the magnetic anomaly source can be simplified as a magnetic dipole [16]. When a magnetic dipole is used as the magnetic anomaly source, the three components of the magnetic field can be expressed as follows:

(1) $$\begin{array}{l} \vec{B}=\frac{\mu_{0}}{4\pi }\left[\frac{3(\vec{m}\cdot \vec{r})\vec{r}}{R^{5}}-\frac{\vec{m}}{R^{3}}\right]\\ =\frac{\mu_{0}}{4\pi R^{5}}\left[\begin{array}{ccc} 3x^{2}-R^{2} & 3xy & 3xz\\ 3xy & 3y^{2}-R^{2} & 3yz\\ 3xz & 3yz & 3z^{2}-R^{2} \end{array}\right]\left[\begin{array}{c} m_{x}\\ m_{y}\\ m_{z} \end{array}\right] \end{array}$$

where $\vec{r}$ represents the relative displacement vector. $R=\left|\vec{r}\right|$ and $\mu_{0}$ is the vacuum permeability.

The magnetic gradient tensor represents the three components of the magnetic field along the three orthogonal directions in space. Usually, the *X*-axis, *Y*-axis, and *Z*-axis in the Cartesian coordinate system are selected, as shown in Equation (2):

(2) $$G=\left[\begin{array}{c} \frac{\partial }{\partial x}\\ \frac{\partial }{\partial y}\\ \frac{\partial }{\partial z} \end{array}\right]\left[\begin{array}{ccc} B_{x} & B_{y} & B_{z} \end{array}\right]=\left[\begin{array}{ccc} B_{xx} & B_{xy} & B_{xz}\\ B_{yx} & B_{yy} & B_{yz}\\ B_{zx} & B_{zy} & B_{zz} \end{array}\right]$$

The magnetic field generated by a magnetic target is stable in space. There is no displacement or conduction current near the magnetic anomaly source and the environmental field is a passive static magnetic field. In this case, according to Maxwell’s equation [15], curl *B* = 0 and div *B* = 0:

(3) $$\{\begin{array}{l} \nabla •B=\frac{\partial B_{x}}{\partial x}+\frac{\partial B_{y}}{\partial y}+\frac{\partial B_{z}}{\partial z}=0\\ \nabla \times B=0 \end{array}$$

By combining Equations (1)–(3), the relationship between the components of the magnetic gradient tensor can be obtained:

(4) $$\{\begin{array}{l} tr(G)=B_{xx}+B_{yy}+B_{zz}=0\\ \begin{array}{l} B_{xy}=B_{yx}\\ B_{xz}=B_{zx}\\ B_{yz}=B_{zy} \end{array} \end{array}$$

Through Equations (3) and (4), Equation (2) can be simplified as follows:

(5) $$G=\left[\begin{array}{ccc} B_{xx} & B_{xy} & B_{xz}\\ B_{xy} & B_{yy} & B_{yz}\\ B_{xz} & B_{yz} & -B_{xx}-B_{yy} \end{array}\right]$$

According to Equation (5), only five of the nine components of the magnetic gradient tensor matrix are independent. According to Equations (1) and (2), the specific calculation formulas of the five independent components can be obtained:

(6) $$\left\{ \begin{array}{l} B_{ij}=\frac{-3\mu_{0}}{4\pi }\frac{\left(\vec{m}-\vec{r})\;(5r_{i}r_{j}-R^{2}\delta_{ij})-R^{2}(r_{i}m_{j}+r_{j}m_{i}\right)}{R^{7}}\\ \delta_{ij}=\left\{ \begin{array}{l} 0,i\neq j\\ 1,i=j \end{array}\right.\;(i,j=1,2,3) \end{array}\right.$$

where 1, 2, and 3 represent *x*, *y*, and *z*, respectively.

## 3. Localization Method

By using the Euler deconvolution formula, Nara et al. proposed the localization method for a magnetic target [13], as shown below:

(7) $$\left[\begin{array}{ccc} B_{\mathrm{xx}} & B_{\mathrm{xy}} & B_{\mathrm{xz}}\\ B_{xy} & B_{yy} & B_{yz}\\ B_{xz} & B_{yz} & B_{zz} \end{array}\right]\left[\begin{array}{c} x\\ y\\ z \end{array}\right]=-3\left[\begin{array}{c} B_{x}\\ B_{y}\\ B_{z} \end{array}\right]$$

According to Equation (7), the relative displacement vector can be obtained:

(8) $$\left[\begin{array}{c} x\\ y\\ z \end{array}\right]=-3{\left[\begin{array}{ccc} B_{\mathrm{xx}} & B_{\mathrm{xy}} & B_{\mathrm{xz}}\\ B_{xy} & B_{yy} & B_{yz}\\ B_{xz} & B_{yz} & B_{zz} \end{array}\right]}^{-1}\left[\begin{array}{c} B_{x}\\ B_{y}\\ B_{z} \end{array}\right]$$

The proposed method by Nara et al. is the simplest localization method for solving the process. Since the right side of the equation requires specific data of the three components of the magnetic field, this method will interfere with the background.

Xu et al. used the difference of two points to replace the three components of the magnetic field and reduce the influence of the background:

(9) $$\left[\begin{array}{c} x_{1}\\ y_{1}\\ z_{1} \end{array}\right]=-{\left(G_{2}-G_{1}\right)}^{-1}\left(G_{2}+3G_{1}\right)\vec{dr}$$

where $G_{1}$ and $G_{2}$ represent the magnetic gradient tensor matrices of the first and second points and $\vec{dr}$ is the relative displacement vector from the second to the first point.

Although this method avoids having to measure three components of the magnetic field, the use of the forward difference method leads to a more significant error in the method.

**Figure 1:**
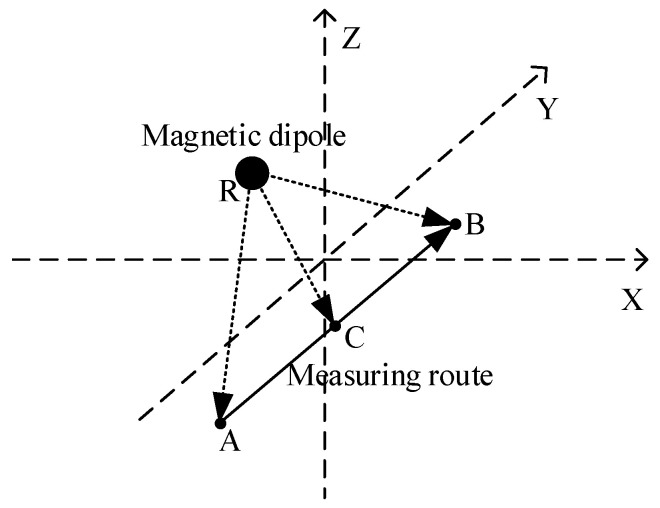
Schematic diagram of the localization method.

The method proposed in this paper is an improvement of the above two methods. The proposed method is as follows: the coordinates of points A, B, and C are known as: ($x_{A}$, $y_{A}$, $z_{A}$), ($x_{B}$, $y_{B}$, $z_{B}$), ($x_{C}$, $y_{C}$, $z_{C}$). Point C is the midpoint of the line between point A and point B. A schematic diagram of the model is shown below (Figure 1):

The magnetic gradient tensors of points A and B are obtained via measurement. The magnetic gradient tensor of point C is obtained with the central difference method. The position of the magnetic dipole can be finally obtained with the tensor relationship of the magnetic gradient of three points and Equation (7), and it is not necessary to measure the three-component value of the magnetic field. $\vec{RA}$ ($x_{RA}$, $y_{RA}$, $z_{RA}$) and $\vec{RB}$ ($x_{RB}$, $y_{RB}$, $z_{RB}$) represent the relative displacement vectors.

(10) $$\left[\begin{array}{c} x_{RB}\\ y_{RB}\\ z_{RB} \end{array}\right]=\left[\begin{array}{c} x_{RA}+\Delta x\\ y_{RA}+\Delta y\\ z_{RA}+\Delta z \end{array}\right]$$

According to Equation (8):

(11) $$\left[\begin{array}{ccc} B_{\mathrm{xxA}} & B_{\mathrm{xyA}} & B_{\mathrm{xzA}}\\ B_{xyA} & B_{yyA} & B_{yzA}\\ B_{xzA} & B_{yzA} & B_{zzA} \end{array}\right]\left[\begin{array}{c} x_{RA}\\ y_{RA}\\ z_{RA} \end{array}\right]=-3\left[\begin{array}{c} B_{\mathrm{xA}}\\ B_{yA}\\ B_{zA} \end{array}\right]$$

(12) $$\left[\begin{array}{ccc} B_{\mathrm{xxB}} & B_{\mathrm{xyB}} & B_{\mathrm{xzB}}\\ B_{xyB} & B_{yyB} & B_{yzB}\\ B_{xzB} & B_{yzB} & B_{zzB} \end{array}\right]\left[\begin{array}{c} x_{RA}+\Delta x\\ y_{RA}+\Delta y\\ z_{RA}+\Delta z \end{array}\right]=-3\left[\begin{array}{c} B_{\mathrm{xB}}\\ B_{yB}\\ B_{zB} \end{array}\right]$$

By subtracting Equations (11) and (12) and using the central difference method, the following formula can be obtained:

(13) $$\left(G_{A}-G_{B}\right)\left[\begin{array}{c} x_{RA}\\ y_{RA}\\ z_{RA} \end{array}\right]=\left(G_{B}+G_{C}\right)\left[\begin{array}{c} \Delta x\\ \Delta y\\ \Delta z \end{array}\right]$$

The relative position vector $\vec{RA}$ can be determined by multiplying both sides of this equation by the $\left(G_{A}-G_{B}\right)$ inverse:

(14) $$\left[\begin{array}{c} x_{RA}\\ y_{RA}\\ z_{RA} \end{array}\right]={\left(G_{A}-G_{B}\right)}^{-1}\left(\left(G_{B}+G_{c}\right)\left[\begin{array}{c} \Delta x\\ \Delta y\\ \Delta z \end{array}\right]\right)$$

(15) $$\left[\begin{array}{c} x_{R}\\ y_{R}\\ z_{R} \end{array}\right]=\left[\begin{array}{c} x_{RA}\\ y_{RA}\\ z_{RA} \end{array}\right]-\left[\begin{array}{c} x_{A}\\ y_{A}\\ z_{A} \end{array}\right]$$

According to Equation (14), at least one of the three variables Δx, Δy, and Δz should not be equal to zero. To reduce the influence of the noise, rotation error, and other errors, a single course measurement is usually carried out so that only one variable changes and the other two variables remain unchanged. The errors caused by the localization method proposed by Xu et al. and this paper are explained in Section 4.

## 4. Simulation

**Figure 2:**
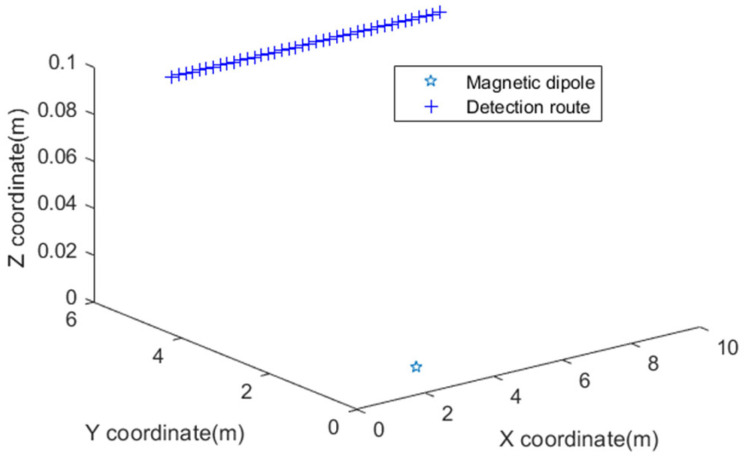
Schematic diagram of magnetic dipole and detection route.

The primary purpose of this section is to describe the testing of the stability and accuracy with the noise. Compared with the results of the other two localization methods, the advantages of the proposed localization method are preliminarily verified. A magnetic dipole is used as a magnetic anomaly source for simulation. The background field is assumed to be a uniform magnetic field. The position coordinate of the magnetic dipole is (3 m, 1 m, 0 m). The detection route is from (1 m, 5 m, 0.1 m) to (8.8 m, 5 m, 0.1 m). The horizontal distance between each measurement is 0.2 m. A schematic diagram of the magnetic dipole and detection route is shown in Figure 2. We refer to Equation (8) as method 1 and Equation (9) as method 2, and the method proposed in this paper is named method 3.

**Figure 3:**
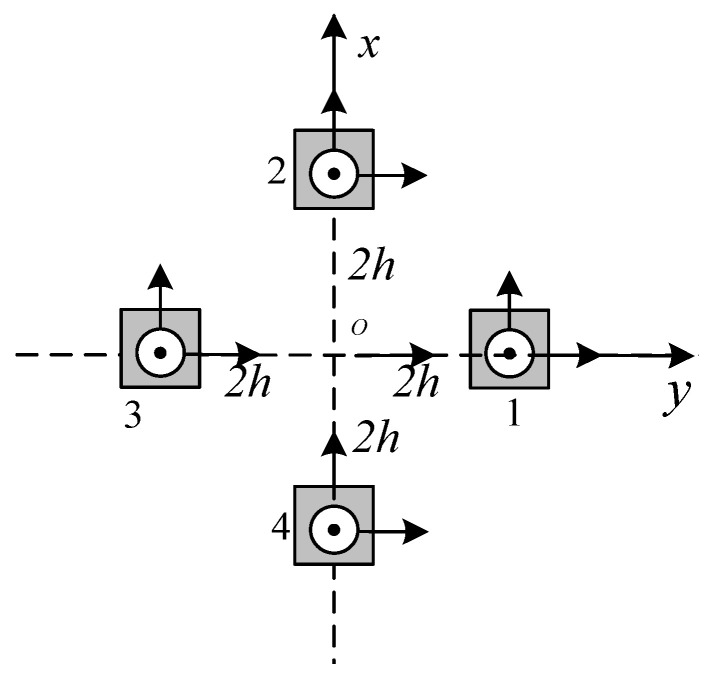
Sensor array.

The sensor array structure is shown in Figure 3:

(16) $$\{\begin{array}{c} B_{xx}=\frac{B_{x}^{1}-B_{x}^{3}}{4h}\\ B_{xy}=\frac{B_{y}^{1}-B_{y}^{3}}{4h}\\ B_{xz}=\frac{B_{z}^{1}-B_{z}^{3}}{4h}\\ B_{yy}=\frac{B_{y}^{2}-B_{y}^{4}}{4h}\\ B_{yz}=\frac{B_{z}^{2}-B_{z}^{4}}{4h} \end{array}$$

**Figure 4:**
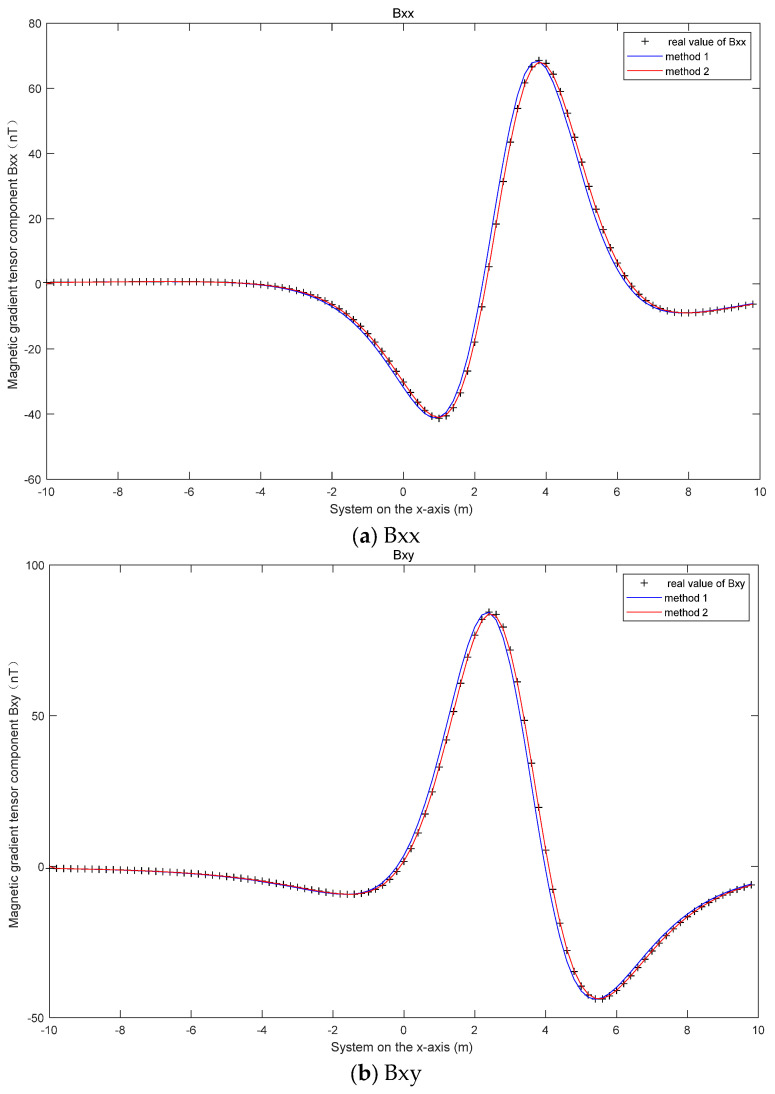

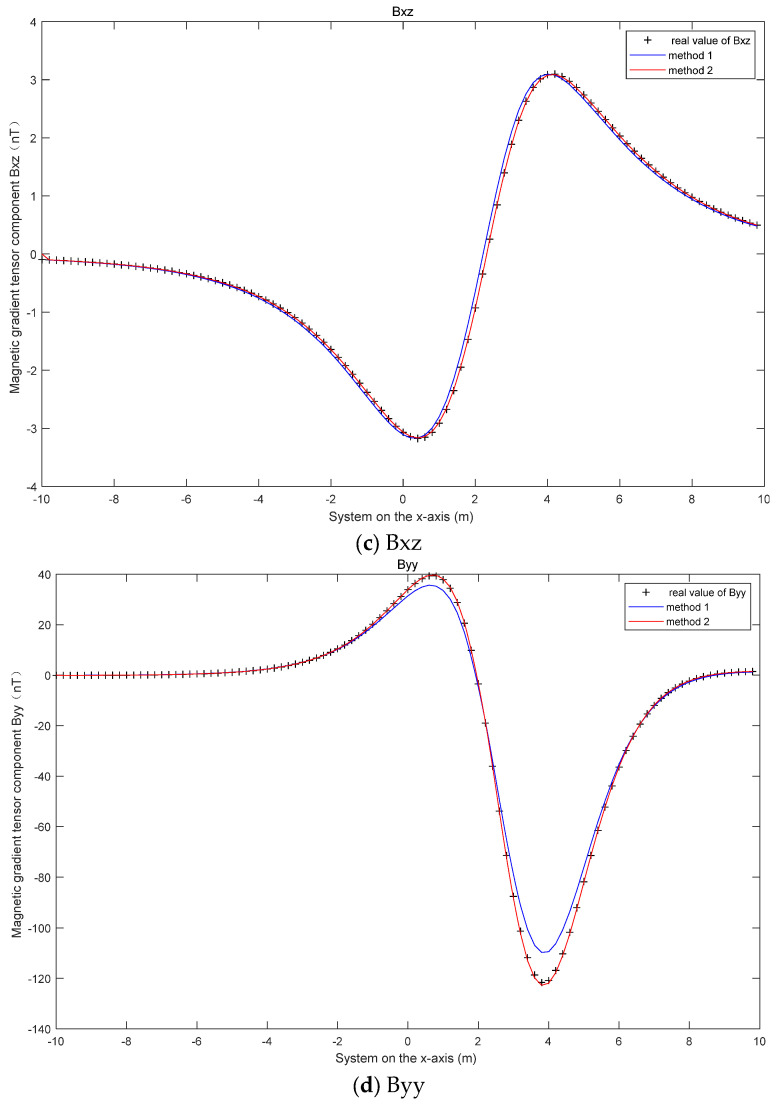

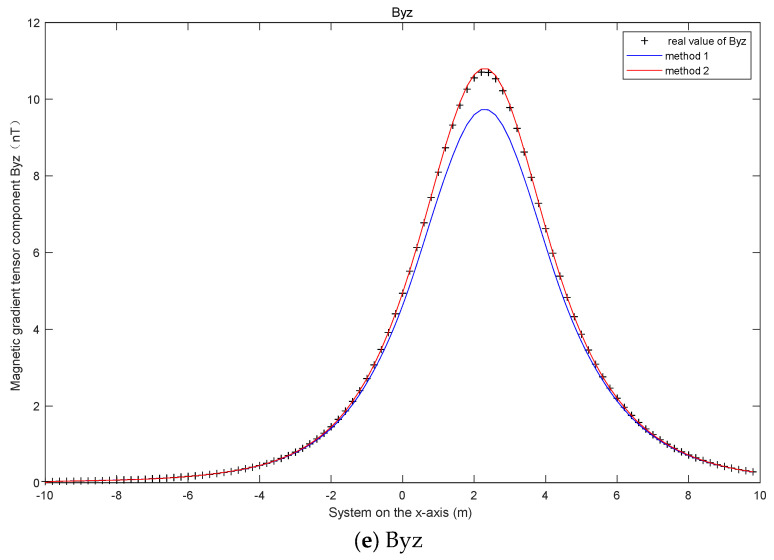
Components of the magnetic gradient tensor.

The value of each component of the magnetic gradient tensor used in the simulation is obtained from Equation (16). The baseline distance is 4 h = 0.4 m. According to the precision required in different situations, we set the maximum allowable error to 0.1 m, 0.15 m, and 0.2 m. The mean absolute error and the RMSE (the root-mean-square-error) are used to analyze the reliability of the various localization methods. The scheme is adopted for the two cases (A) and (B).

(A) The magnetic moments of the magnetic dipole are $\vec{m}$ (50 $A\cdot m^{2}$, 30 $A\cdot m^{2}$, 10 $A\cdot m^{2}$). Gaussian white noise with a signal-to-noise ratio of 62 dB is added to the measured magnetic field. By comparing the errors of the magnetic gradient tensor, the accuracy of methods 2 and 3 can be compared, as shown in Figure 4.

**Figure 5:**
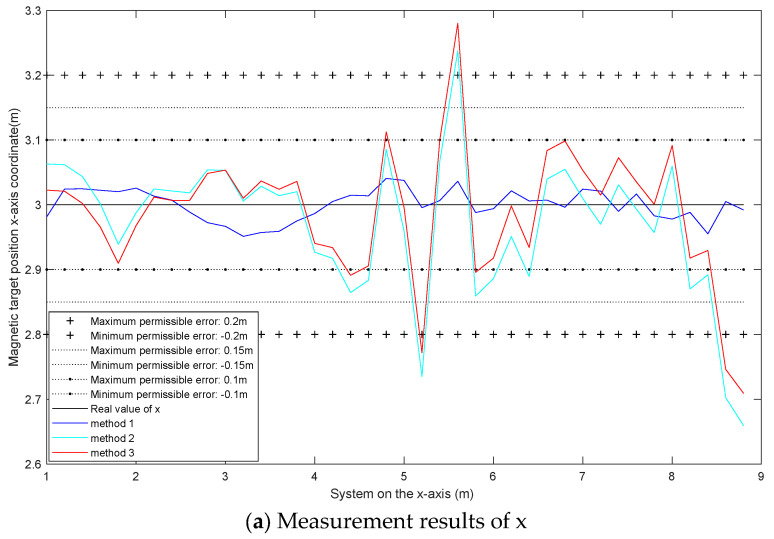

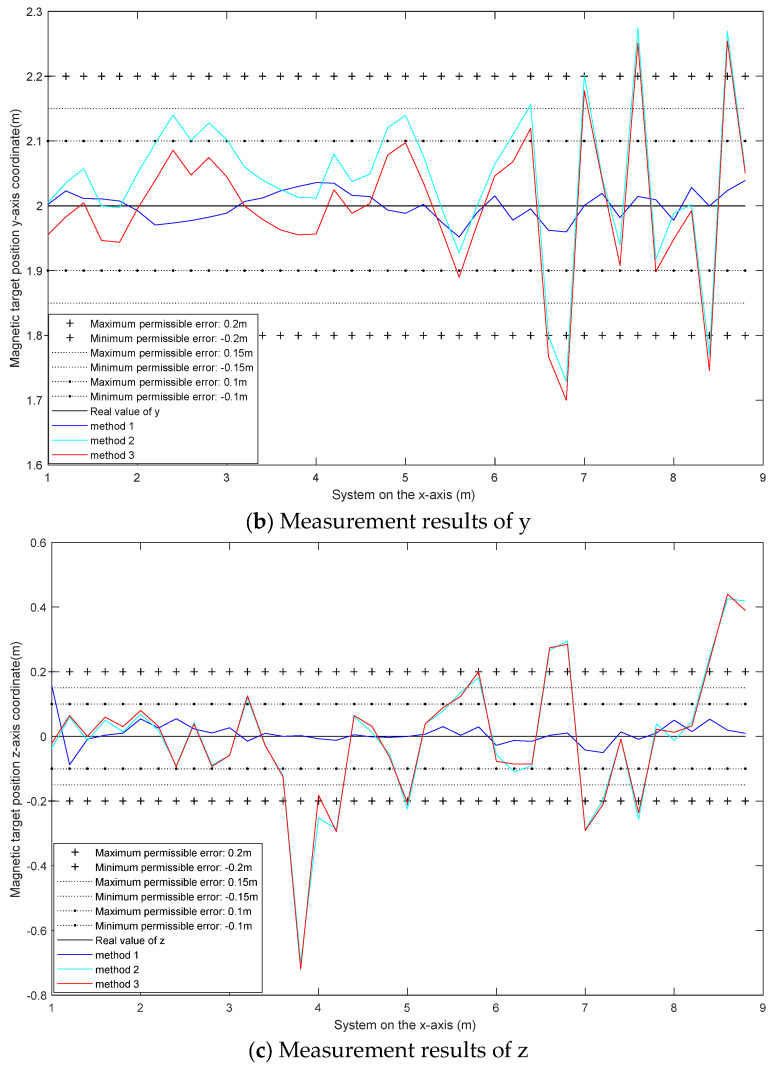
Localization result diagram with 62 dB Gaussian white noise.

The principal formula of the forward difference method is shown in Equation (17). For contrast, the main formula is illustrated in Equation (18).

(17) $$f^{\prime }(x)=\frac{f(x+\Delta x)-f(x)}{\Delta x}-\frac{\Delta x}{2!}f^{\prime \prime }(x)-\ldots$$

(18) $$f^{\prime }(x)=\frac{f(x+\Delta x)-f(x-\Delta x)}{2\Delta x}-\frac{{\left(\Delta x\right)}^{3}}{3!}f^{\left(3\right)}(x)+\ldots$$

(B) The magnetic moments of the magnetic dipole are $\vec{m}$ (50 $A\cdot m^{2}$, 30 $A\cdot m^{2}$, 10 $A\cdot m^{2}$). Gaussian white noise with a signal-to-noise ratio of 62 dB is added to the measured magnetic field. The localization results are shown in Figure 5.

The localization data for the above two experiments are analyzed using the MAE (mean absolute error) and the RMSE. The calculation results are shown in Table 1 and Table 2. The calculation formula of RMSE is shown in Equation (19). The MAE formula is shown in Equation (20), where $\eta_{r}$ is the coordinate estimate, $\eta_{ei}$ is the actual value, and N is the number of the point.

(19) $$E_{RMS}=\sqrt{\sum_{i=1}^{N}{\left(\eta_{r}-\eta_{ei}\right)}^{2}/N}$$

(20) $$M_{AE}=\sum_{i=1}^{N}(\eta_{r}-\eta_{ei})/N$$

Figure 4 shows the results of the approximate measurement of the five magnetic gradient tensor components with the forward difference method and the central difference method. It can be seen from Figure 4 that the error of the magnetic gradient tensor calculated with the central difference method is smaller than that obtained with the forward difference method. This also verifies the correctness of Equations (17) and (18).

It can be seen from Figure 5 that the localization error of method 1 is smaller. The error of the localization results of each measurement point in method 1 is mostly within 0.1 m. The error of the localization results of each measurement point of method 3 is smaller than that of method 2. The localization errors of methods 2 and 3 at each measurement point are mostly within 0.2 m. As shown in Table 1 and Table 2, with only noise interference, method 3 is more accurate than method 2, but slightly less accurate than method 1. Method 3 is more accurate than method 2 because method 2 uses the forward difference method to approximate the magnetic gradient tensor, while method 3 uses the central difference method to solve the magnetic gradient tensor. Method 3 is slightly less accurate than method 1 because more points are used, leading to larger errors in solving the inverse matrix.

The above conclusion is the simulation for the condition of simple noise. During measurement, due to the existence of the geomagnetic field and various complex interferences, the three components of the magnetic dipole’s own magnetic field are submerged, resulting in measurement errors. In order to verify whether method 1 becomes larger in an experiment and whether the error of the method proposed in this paper is still small, an experiment is carried out.

## 5. Experiment

To verify the accuracy of the localization results using the proposed method under complex magnetic interference conditions, the following experiments are carried out:

The detection process is described below.

**Figure 6:**
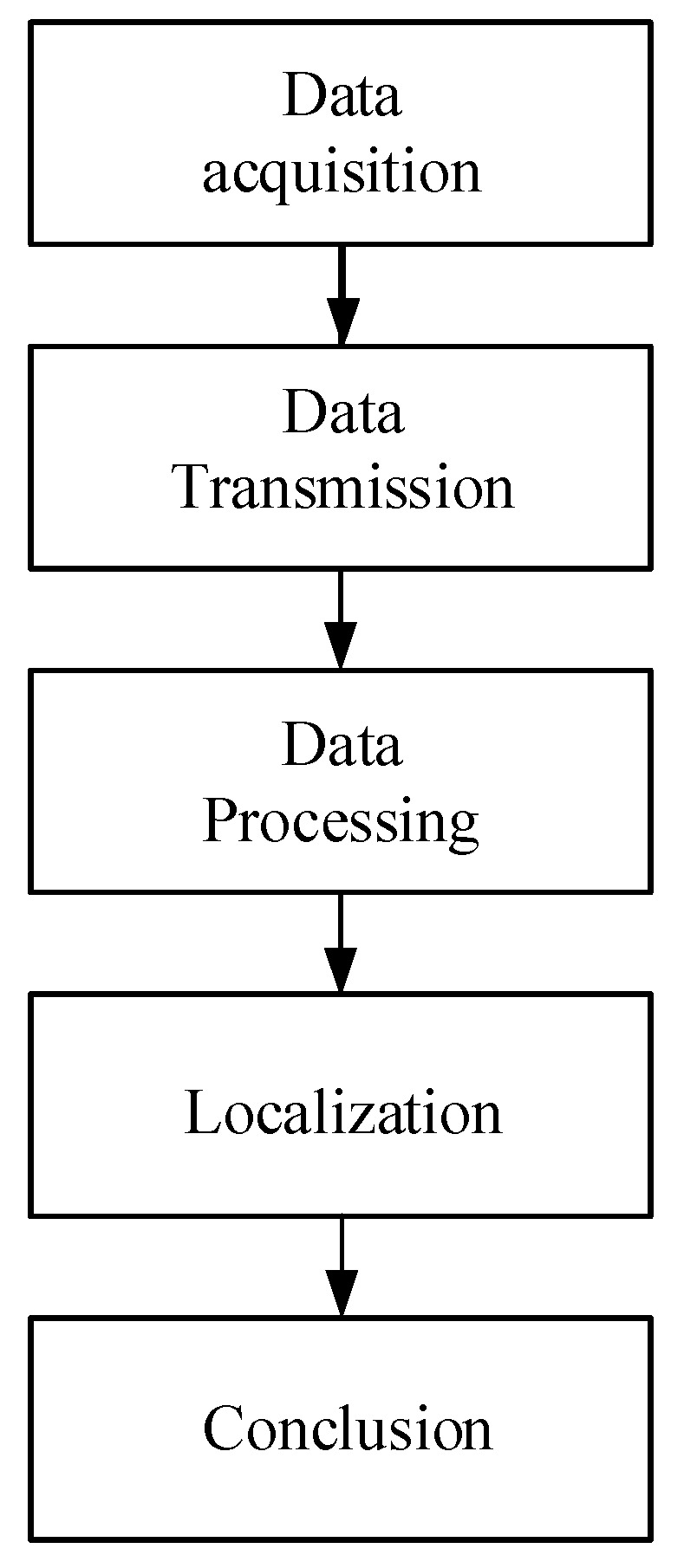
Detection flowchart.

**Figure 7:**
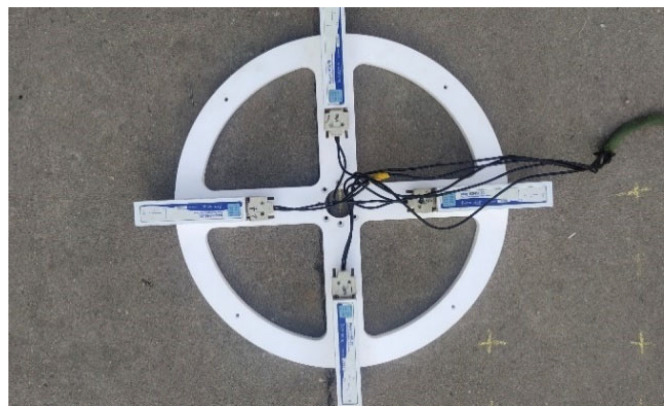
Magnetic gradient tensor detection device.

This detection process is described in Figure 6. The detection device is shown in Figure 7. The device consists of four three-axis fluxgate sensors and a nonmagnetic chassis. The operating temperature range of the sensor is −40~70 °C. The measurement range of the sensor is 70,000 nt. The maximum resolution of the sensor is 0.01 nt. The baseline distance is 0.4 m.

When the magnetic anomaly source is detected, the sensor transmits the data to the data acquisition card through the data transmission line, and the data is processed by the information processing terminal. The results are obtained according to the corresponding algorithm. The flowchart is shown in the following figure. 

The experimental site is a cement road surface with complex magnetic interference. As in the simulation, we refer to Equation (8) as method 1 and Equation (9) as method 2, and the method proposed in this paper is called method 3.

**Figure 8:**
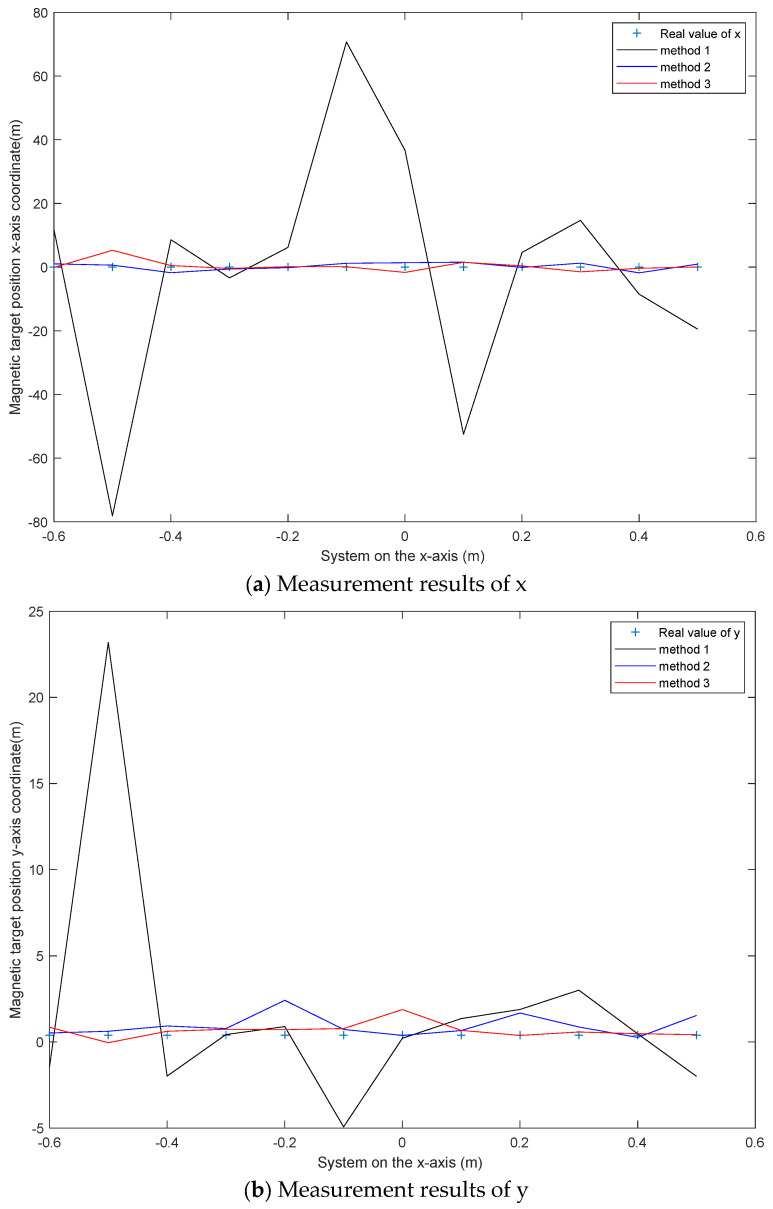

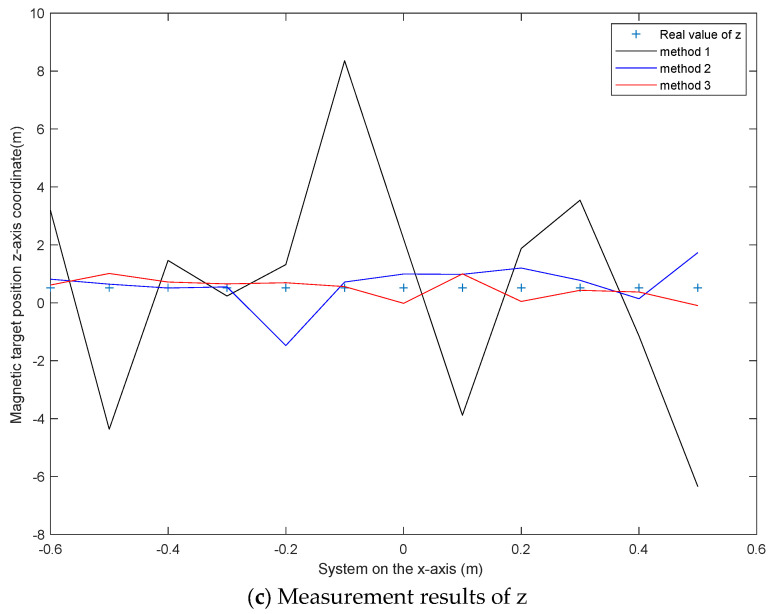
Localization result diagram.

**Figure 9:**
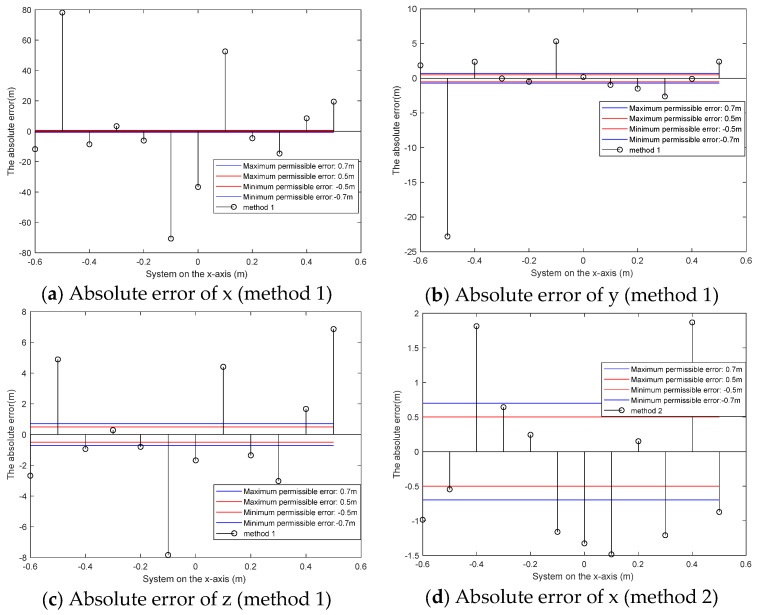

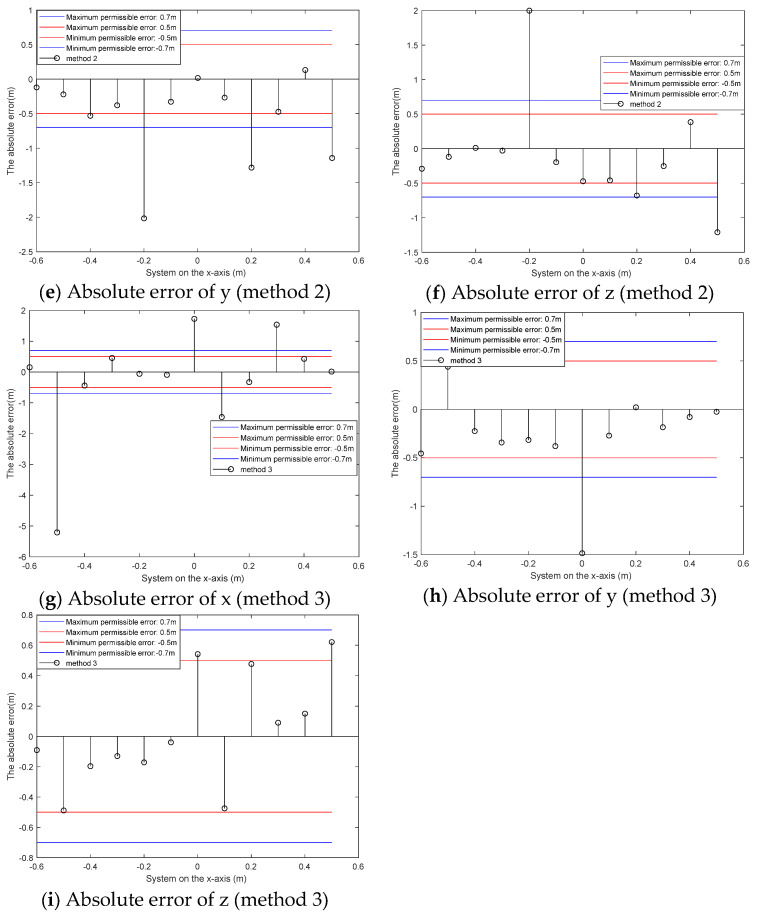
Absolute error of the localization result.

A cylindrical magnet with a bottom diameter of 0.02 m and a height of 0.02 m is the magnetic anomaly source for detection. The detection path is from (−0.6 m, 0.8 m, 0.6 m) to (0.5 m, 0.8 m, 0.6 m) and the distance between detection points is 0.1 m. Cylindrical magnets are placed at (0.05 m, 0.4 m, 0.52 m). Unlike the simple noise conditions in the simulation, the interference in the field experiment is complex, and the localization error increases. This section describes how we study the reliability of the localization results of the three methods with the allowable errors of 0.5 m and 0.7 m. The localization results are shown in Figure 8. The absolute error diagram of each point is shown in Figure 9. There are twelve measuring points shown in Figure 8. Table 3 shows the error measured for each point. The RMSE of the localization results is shown in Table 4. The acceptance probability is used to evaluate the localization results of various methods. The results are shown in Table 5 and Table 6.

It can be seen from Figure 8 that the error of method 3 is smaller than those of method 1 and method 2 under the condition of complex magnetic interference. The error of method 1 with a smaller error in the simulation becomes larger sharply in the condition. The reason for this is that its localization method needs to measure three components of the geomagnetic field, but there are errors in the measurement of the three components of the geomagnetic field under complex magnetic interference conditions.

Figure 9 and Table 3 give the absolute error of each point. It can be seen from the chart that the overall error of method 3 is smaller. It can be determined from Table 5 and Table 6 that the localization result of method 3 is more reliable with the premise of error ranges of 0.5 m and 0.7 m. Table 4 shows the deviation between the localization results of the three methods and the true value. The results show that the deviation of the third method is smaller.

From the above figures and tables, the following conclusions can be drawn:

(1) The accuracy of method 1 is worse than those of method 2 and method 3 under complex magnetic interference conditions;
(2) The accuracy of method 3 is higher than that of method 2;
(3) The accuracy of method 3 is higher than that of method 2, which also proves the correctness of Equations (17) and (18). It is also proved that using the central difference method to measure the magnetic gradient tensor makes the data error smaller.

## 6. Conclusions

In this paper, a small object localization method based on a magnetic gradient tensor is proposed. The three components of the magnetic field to be measured are transformed into the magnetic gradient tensor matrix at the midpoint using the center difference method to reduce the interference of the background field. The algorithm is simple and does not involve time-consuming methods, such as iteration, which makes the method discussed in this paper real-time. Simulation results show the reliability of the proposed method only under noise interference. The experimental results show that this method is superior to the other two localization methods under complex magnetic interference conditions. In practice, complex magnetic interference often leads to measurement errors. Complex magnetic interference includes the geomagnetic field, magnetic field of surrounding ferromagnetic objects, the noise of the instrument itself, etc. Determining how to reduce the influence of such interference and get real-time positioning results is the purpose of this research. In the next step, the work of this research will be continued, and the influence of complex magnetic interference will be reduced by changing the measurement method of the magnetic gradient tensor, optimizing the instrument calibration, and upgrading the noise reduction algorithm.

## Figures and Tables

**Table 1:** Mean absolute error of the localization results for each component.

Method	Mean Absolute Error (m)		
	X	Y	Z
method 1	2.5036 × 10^−4^	−4.5075 × 10^−4^	0.0086
method 2	−0.00291	0.0402	−0.0089
method 3	−0.0112	−1.7783 × 10^−4^	−0.0054

**Table 2:** RMSE of the localization results for each component.

Method	X	Y	Z
method 1	0.0235	0.0220	0.0371
method 2	0.1117	0.1168	0.2003
method 3	0.1024	0.1095	0.1994

**Table 3:** The absolute error of the localization results of each component.

Sets	Method 1			Method 2			Method 3		
	X (m)	Y (m)	Z (m)	X (m)	Y (m)	Z (m)	X (m)	Y (m)	Z (m)
1	−11.7927	1.8625	−2.6758	−0.9852	−0.1208	−0.2911	0.1531	−0.4555	−0.0901
2	78.1653	−22.7930	4.8900	−0.5452	−0.2214	−0.1194	−5.2043	0.4403	−0.4885
3	−8.5264	2.3832	−0.9387	1.8135	−0.5309	0.0114	−0.4386	−0.2244	−0.1963
4	3.4051	−0.0306	0.2861	0.6430	−0.3781	−0.0284	0.4566	−0.3412	−0.1297
5	−6.1177	−0.4957	−0.7971	0.2428	−2.0167	1.9977	−0.0568	−0.3159	−0.1711
6	−70.6470	5.3333	−7.8377	−1.1601	−0.3289	−0.1962	−0.0905	−0.3785	−0.0391
7	−36.6464	0.1855	−1.6782	−1.3265	0.0166	−0.4725	1.7255	−1.4845	0.5409
8	52.5325	−0.9614	4.4035	−1.4856	−0.2676	−0.4592	−1.4613	−0.2707	−0.4745
9	−4.5710	−1.4868	−1.3525	0.1489	−1.2822	−0.6792	−0.3275	0.0209	0.4761
10	−14.6448	−2.6086	−3.0219	−1.2083	−0.4735	−0.2534	1.5386	−0.1843	0.0900
11	8.5505	−0.0691	1.6672	1.8666	0.1320	0.3830	0.4270	−0.0781	0.1500
12	19.5017	2.3970	6.8638	−0.8735	−1.1424	−1.2102	0.0138	−0.0249	0.6206

**Table 4:** RMSE of usable points.

	X (m)	Y (m)	Z (m)
method 1	127.0947	23.9393	13.3102
method 2	4.0090	2.8155	2.5886
method 3	5.9406	1.7691	1.2220

**Table 5:** Analysis of usable points (maximum error: 0.5 m).

	Method 1			Method 2			Method 3		
	X	Y	Z	X	Y	Z	X	Y	Z
Number of acceptable points	0	4	1	2	8	9	8	11	10
Acceptable probabilities	0	33.3%	8.3%	16.7%	66.7%	75%	66.7%	91.7%	83.3%

**Table 6:** Analysis of usable points (maximum error: 0.7 m).

	Method 1			Method 2			Method 3		
	X	Y	Z	X	Y	Z	X	Y	Z
Number of acceptable points	0	5	1	4	9	10	8	11	12
Acceptable probabilities	0	41.7%	8.3%	33.3%	75.0%	83.3%	66.7%	91.7%	100.0%

## Data Availability

All the data in this paper can be obtained from the first author.

## References

[1] Li Q., Li Z., Zhang Y., Yin G. (2018). Artificial vector calibration method for differencing magnetic gradient tensor systems. Sensors.

[2] Beran L., Oldenburg D.W. (2008). Selecting a discrimination algorithm for unexploded ordnance remediation. IEEE Trans Geosci. Remote Sens..

[3] Karimi K., Shirzaditabar F. (2017). Using the ratio of the magnetic field to the analytic signal of the magnetic gradient tensor in determining the position of simple shaped magnetic anomalies. J. Geophys. Eng..

[4] Song S., Li B., Qiao W., Hu C., Ren H., Yu H., Zhang Q., Meng M.Q.-H., Xu G. (2014). 6-D magnetic localization and orientation method for an annular magnet based on a closed-form analytical model. IEEE Trans. Magn..

[5] Wang X., Liu H., Wang H., Ge J., Dong H., Liu Z. (2020). Quantitative Analysis of the Measurable Areas of Differential Magnetic Gradient Tensor Systems for Unexploded Ordnance Detection. IEEE Sens. J..

[6] Xiu C., Meng X., Guo L., Zhang S., Zhang X. (2018). Compensation for aircraft effects of magnetic gradient tensor measurements in a towed bird. Explor. Geophys..

[7] Song Q., Ding W., Peng H., Shuai J., Wang B. (2017). A new magnetic testing technology based on magnetic gradient tensor theory. Insight-Non-Destr. Test. Cond. Monit..

[8] Connolly P.R.J., Yan W., Zhang D., Mahmoud M., Verrall M., Lebedev M., Iglauer S., Metaxas P.J., May E.F., Johns M.L. (2019). Simulation and experimental measurements of internal magnetic field gradients and NMR transverse relaxation times(T2) in sandstone rocks. J. Pet. Sci. Eng..

[9] Sui Y., Xia Z., Zhou Z., Wang Y., Lin J. (2018). A ground-based test facility for airborne magnetic gradient tensor instruments simulating calibration flights. Measurement.

[10] Hu S., Tang J., Ren Z., Chen C., Zhou C., Xiao X., Zhao T. (2018). Multiple underwater objects localization with magnetic gradiometry. IEEE Geosci. Remote Sens. Lett..

[11] Zhi H., Ma T., Pei D., Sun H. (2020). A novel magnetic dipole inversion method based on tensor geometric invariants. AIP Adv..

[12] Yin G., Zhang Y., Li Z., Fan H., Ren G. (2016). Detection of ferromagnetic target based on mobile magnetic gradient tensor system. J. Magn. Magn. Mater..

[13] Nara T., Suzuki S., Ando S. (2006). A closed-form formula for magnetic dipole localization by measurement of its magnetic field and spatial gradients. IEEE Trans. Magn..

[14] Yin G., Zhang Y., Fan H., Li Z. (2014). Magnetic dipole localization based on magnetic gradient tensor data at a single point. J. Appl. Remote Sens..

[15] Wang B., Ren G., Li Z., Li Q. (2021). A third-order magnetic gradient tensor optimization algorithm based on the second-order improved central difference method. AIP Adv..

[16] Wang B., Ren G., Li Z., Li Q. (2021). The stability optimization algorithm of second-order magnetic gradient tensor. AIP Adv..

[17] Xu L., Gu H., Chang M., Fang L., Lin P., Lin C. (2021). Magnetic Target Linear Localization Method Using Two-Point Gradient Full Tensor. IEEE Trans. Instrum. Meas..

